# Purification, Chemical Characterization and Immunomodulatory Activity of a Sulfated Polysaccharide from Marine Brown Algae *Durvillaea antarctica*

**DOI:** 10.3390/md20040223

**Published:** 2022-03-24

**Authors:** Ling Qin, Hui Xu, Yingying He, Chen Liang, Kai Wang, Junhan Cao, Changfeng Qu, Jinlai Miao

**Affiliations:** 1 Key Laboratory of Marine Eco-Environmental Science and Technology, First Institute of Oceanography, Ministry of Natural Resource, Qingdao 266061, China; qinling@fio.org.cn (L.Q.); xuhui@fio.org.cn (H.X.); heyinging@fio.org.cn (Y.H.); liangchen@fio.org.cn (C.L.); wk2303@stu.ouc.edu.cn (K.W.); caojunhan@fio.org.cn (J.C.); 2 Laboratory for Marine Drugs and Bioproducts, Qingdao Pilot National Laboratory for Marine Science and Technology, Qingdao 266237, China; 3 Marine Natural Products R&D Laboratory, Qingdao Key Laboratory, Qingdao 266061, China

**Keywords:** *Durvillaea antarctica*, fucoidan, structural characterization, immunomodulatory

## Abstract

An immunomodulatory polysaccharide (DAP4) was extracted, purified, and characterized from *Durvillaea antarctica.* The results of chemical and spectroscopic analyses demonstrated that the polysaccharide was a fucoidan, and was mainly composed of (1→3)-α-l-Fuc*p* and (1→4)-α-l-Fuc*p* residues with a small degree of branching at C-3 of (1→4)-α-l-Fuc*p* residues. Sulfate groups were at C-4 of (1→3)-α-l-Fuc*p*, C-2 of (1→4)-α-l-Fuc*p* and minor C-6 of (1→4)-β-d-Gal*p*. Small amounts of xylose and galactose exist in the forms of β-d-Xyl*p*-(1→ and β-d-Gal-(1→. The immunomodulatory activity of DAP4 was measured on RAW 264.7 cells, the results proved that DAP4 exhibited excellent immunomodulatory activities, such as promoted the proliferation of spleen lymphocytes, increased NO production, as well as enhanced phagocytic of macrophages. Besides, DAP4 could also produce better enhancement on the vitality of NK cells. For the high immunomodulatory activity, DAP4 might be a potential source of immunomodulatory fucoidan with a novel structure.

## 1. Introduction

Seaweeds are rich in nutrients and bioactive compounds, sulfated polysaccharides are not only an important part of the cell wall, but also an important component with various biological activities. At present, sulfated polysaccharide is widely used in the food and pharmaceutical industries as it could be used as nutritional ingredient or therapeutic agent [1,2,3]. The algae polysaccharide possessed various biological activities, such as anti-tumor, immunomodulatory, anticoagulant, antioxidant, and anti-inflammatory [3,4,5,6]. Fucoidan is a kind of sulfated polysaccharide, consisting mainly of fucose and sulfate groups. Fucoidan was usually extracted from brown algae, sea cucumbers, and sea urchins. Commonly, fucoidan contains the main chain composed by 1,3-linked α-L-fucose residues, or a backbone consisted of alternating 1,3- and 1,4-linked α-L-fucose residues, with sulfate groups often occupying either the C-2, C-3, C-4, or a combination, positions [7]. Besides, fucoidan derived from brown alga is a complex branched polysaccharide, the side chains usually contain mannose, galactose, glucose, xylose, and uronic acid [8,9].

The immune system is the most effective defender against various pathogenic microbial infections before the adaptive immune system is activated [10]. It maintains the normal operation of the body and plays a vital role in human health through various cell types and a multitude of secreted factors [11,12]. The occurrence of many diseases, such as malignancies, bacterial, or viral infections, is closely related to immune disorders or immune deficiency. Sulfated polysaccharide has recently been cited as a potent immunomodulatory agent for treating these diseases [13].

Fucoidan has few side effects and has been proved to be a potential natural drug candidate in literature; besides, brown algae and marine invertebrates are the main sources of fucoidan [14]. *Durvillaea antarctica*, also known as cochayuyo and rimurapa, distributed along the coasts of Chile, southern New Zealand and Macquarie Island [15,16], is used in cuisine, and it also has medicinal values. *D. antarctica* typically contains around 11% protein, 65% carbohydrates, and 0.8–4.3% total lipids [17]. As previously reported, a soluble glucan extracted from *D. antarctica* has immunomodulatory activities, antioxidant activity, anti-viral activities, and anti-tumor activities, and the glucan consists of β-1,3- and β-1,6-glucose residues [18,19,20,21]. However, there are few reports about fucoidans extracted from *D. Antarctica.* A novel fucoidan isolated and purified from *D. Antarctica* could effectively promote the growth of leukocytes in the treatment of chemotherapy-treated mice was reported [22]. Up to date, there are few records of fucoidan prepared from *D. Antarctica*, its structural characteristics and biological activities have not been systematically studied. In the present work, we isolated and purified the fucoidan from brown algae *D. Antarctica*, and the structural characteristics were determined by methylation analysis and NMR spectrometry analysis. At the same time, its potential immunomodulatory activity in vitro was evaluated, including lymphocyte proliferation, phagocytic of macrophages, NO production, and NK cell cytotoxicity in vitro.

## 2. Results and Discussion

### 2.1. Chemical Characteristics of the Sulfated Polysaccharide DAP4

**Figure 1:**
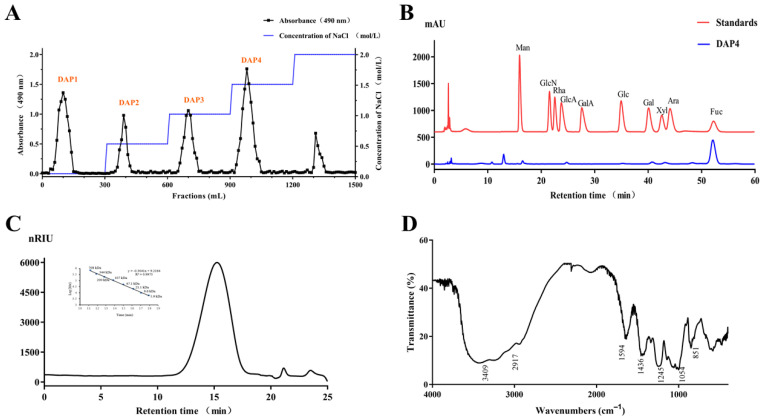
Q Sepharose Fast Flow column, HPLC chromatograms, and IR spectrum of DAP4. (**A**) The crude polysaccharide was separated by a Q Sepharose Fast Flow column.; (**B**) monosaccharide composition analysis of DAP4; (**C**) HPGPC chromatogram of DAP4 and the standard curve of molecular weight; and (**D**) IR spectrum of DAP4.

The crude polysaccharide was extracted from *D. antarctica* with hot water, the yield of fucoidan was 17.52% and designed as DAP. Then, the DAP was divided into four fractions by using a Q Sepharose Fast Flow column (Figure 1A). The chemical compositions of DAP and DAP1 to DAP4 were shown in Table 1. Composition analysis suggested that all fractions were heterogeneous polysaccharides, there were differences in the monosaccharide ratios, the molecular weight, and the degree of sulfation. DAP4 was further purified by gel filtration chromatography on a Sephacryl S-300/HR column and obtained as the major fraction. Compared with other fractions, DAP4 holds the highest sulfate content (22.3%) and fucose content (87.9%). As shown in Figure 1B, DAP4 contained fucose (Fuc), xylose (Xyl), galactose (Gal), and mannose (Man). DAP4 appeared as a single and symmetrical peak in the HPGPC chromatogram (Figure 1C), its average molecular weight was about 92 kDa according to its retention time in the HPGPC chromatogram. Like previous reports, fucoidan derived from brown seaweeds was usually combined with galactose, mannose, and uronic acids, in the form of galactofucan/fucogalactan, fucoglucuronomannan/glucuronofucans, and more complex heteropolysaccharide [7,23,24]. Thus, the fucoidan fraction DAP4 isolated from *Durvillaea antarctica* has a typical characteristic of fucoidan composition.

There are some characteristic peaks of functional groups in the FTIR spectrum (Figure 1D) of DAP4. The peaks at 3409 and 1054 cm^−1^ were from stretching vibration of O–H and C–O, respectively [25]. The peaks at 851 and 1245 cm^−1^ derived from the bending vibration of C–O–S of sulfate in axial position and stretching vibration of S–O of sulfate [26,27], respectively, suggesting the presence of sulfate ester in DAP. In addition, the peak at 1594 cm^−1^ was due to the bending vibration of O–H, the signal at 2917 cm^−1^ was due to the stretching vibration of C–H [28]. The peak at 1436 cm^−1^ corresponded to the absorption peak of variable angle vibration of the C–H bond.

### 2.2. Structural Characterization of DAP4

#### 2.2.1. Methylation Analysis

Methylation analysis of DAP4 and its desulfated product DAP4 could provide information of the linkage pattern and sulfation position in DAP4. The results of the methylation analysis of DAP4 and DAP4-Ds are listed in Table 2. The results showed that the 2,4-di-O-methylfucitol, 2,3-di-O-methylfucitol, and 2-O-methylfucitol account for the main partially methylated alditol acetates, indicating that the major linkage type of DAP4 was (1→3), (1→4), and (1→3,4)-linked fucose units, respectively. After desulfation, the molecular ratio of 2,4-di-O-methylfucitol and 2,3-di-O-methylfucitol increased from 24.73% and 21.42% to 37.92% and 28.23%, respectively, while the amounts of 2-O-methylfucitol and 3-O-methylfucitol decreased drastically. Thus, it could be concluded that the sulfate esters were mainly substituted at the C-4 positions of the (1→3)-linked fucose unit and C-2 positions of (1→4)-linked fucose unit according to the variation of the proportions of the methylated derivatives. Furthermore, a certain amount of 2,3,4-tri-methylfucitol was detected, indicating the presence of terminal fucose units, and a minor amount of terminal xylose and galactose units, (1→4) and (1→4,6)-linked galactose units were also observed. Besides, the disappearance of (1→4,6)-linked galactose units in DAP4-Ds suggested that the sulfate ester might substitute at C-6 of (1→4)-linked galactose units. Total ion current chromatograms and MS spectrum of DAP4 and DAP4-Ds on GC-MS are shown in Figure S1A,B. The above results suggested that the linkages of DAP4 were mainly constituted of 3-linked α-l-fucopyranose residues, 4-linked α-l-fucopyranose residues, and little 4-linked β-d-galactopyranose residues, partially sulfated groups were at C-4 of (1→3)-linked fucose residues, C-2 of (1→4)-linked fucose residues, and C-6 of (1→4)-linked galactose residues. The fine structure was further confirmed by NMR.

#### 2.2.2. NMR Analysis

**Figure 2:**
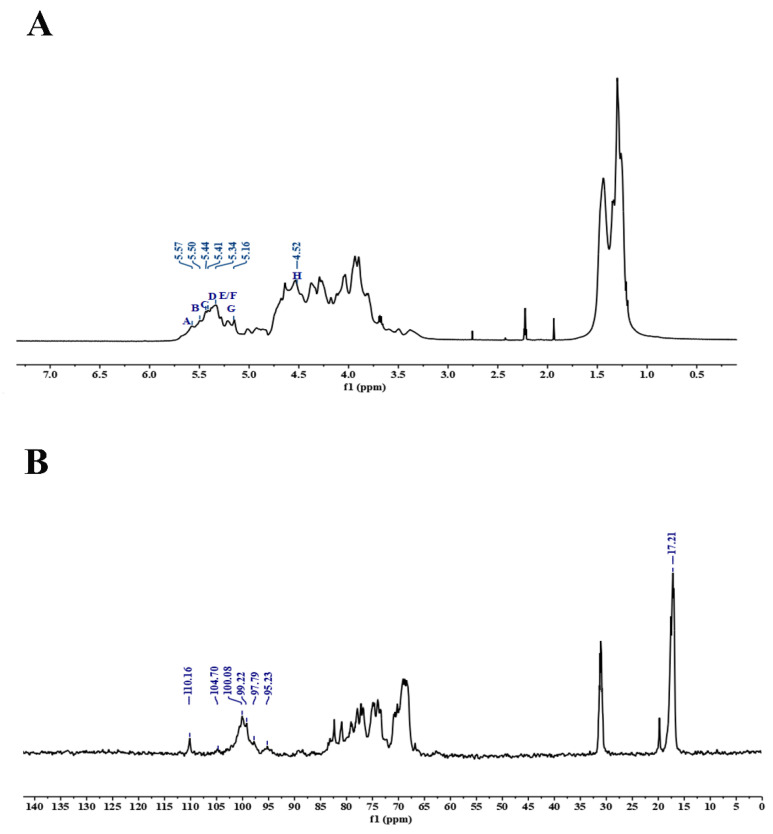

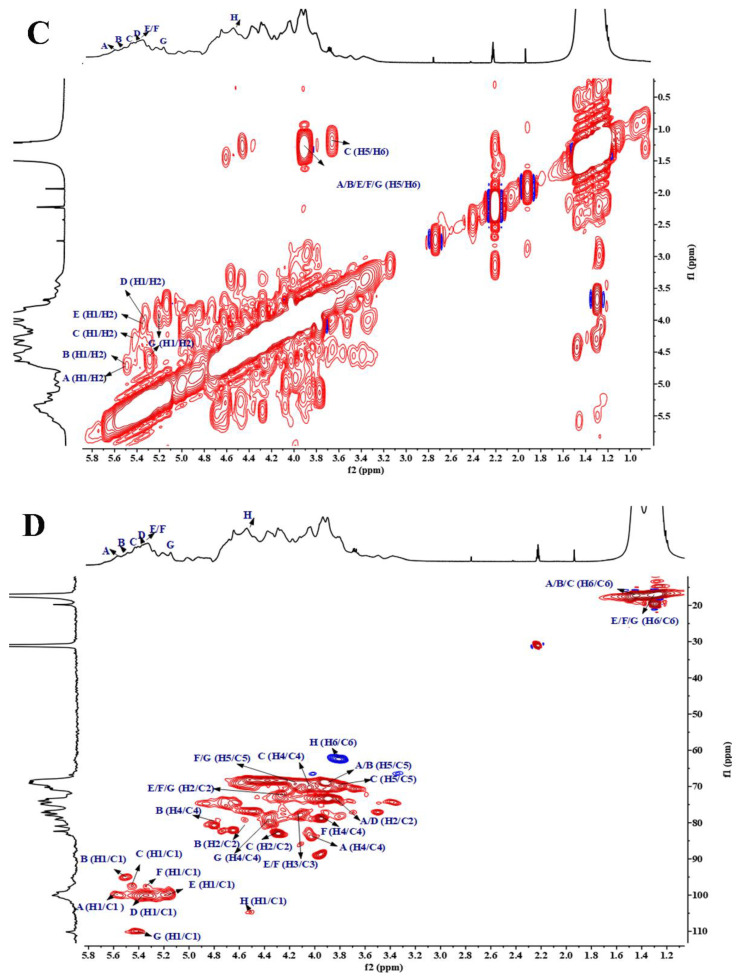

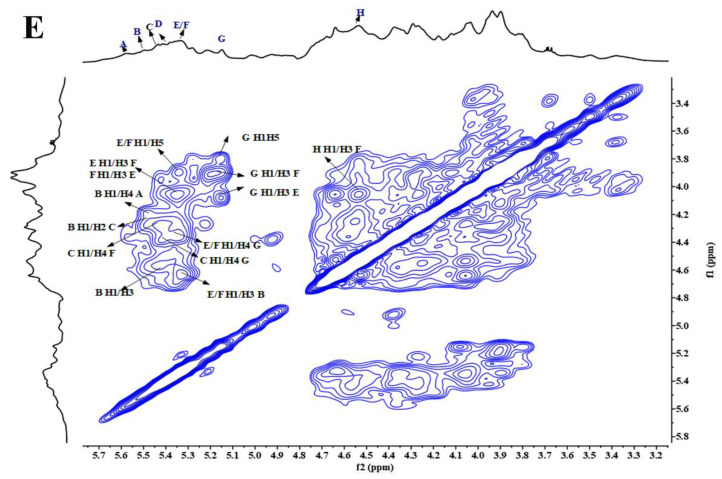
NMR spectra of DAP4. Spectra were performed on an Agilent DD2 500 MHz NMR spectrometer using acetone as internal standard. (**A**) ^1^H NMR spectrum; (**B**) ^13^C spectrum; (**C**) ^1^H–^1^H COSY spectrum; (**D**) ^1^H–^13^C HSQC spectrum; (**E**) ^1^H–^1^H NOESY spectrum. A–H correspond to →4)-α-l-Fuc*p*(2SO_4_)-(1→, →3)-α-l-Fuc*p*(4SO_4_)-(1→, →2)-α-l-Fuc*p*-(1→, β-d-Xyl*p*-(1→, →3)-α-l-Fuc*p*-(1→, →3,4)-α-l-Fuc*p*-(1→, →4)-α-l-Fuc*p*-(1→ and β-d-Gal*p*-(1→.

The structural features of the polysaccharide DAP4 were further analyzed by 1D and 2D NMR spectra. In the ^1^H NMR spectrum of DAP4 (Figure 2A), the anomeric proton signals at 5.57, 5.50, 5.44, 5.34, and 5.16 ppm (labeled as A–F) were assigned to α-l-fucopyranose residues [29,30], while the high-field signal at 4.52 ppm (labeled as H) was assigned as β-d-galactopyranose residue [31]. Strong signals at 1.30 and 1.43 ppm were characteristic of H-6 of fucose residues, and the chemical shifts ranging from 4.70 to 3.60 ppm were designated as the protons present on C2–C6. In the ^13^C NMR spectrum (Figure 2B), the signals at 100.08, 99.20, 97.79, and 95.23 ppm were assigned to be anomeric carbon signals of fucose residues; the anomeric carbon signals at 104.70 and 110.16 ppm were assigned to galactose and terminal xylose residue, respectively. The signals at 62–83 ppm were attributed to the C-2−C-6 of the sugar residues. Moreover, the resonation of −CH_3_ at 17.22 ppm indicated the C-6 of the fucose residue.

The assignments of signal in ^1^H and ^13^C NMR spectra were carried out using two dimensional homonuclear ^1^H–^1^H COSY (Figure 2C), ^1^H–^1^H NOESY (Figure 2E), and heteronuclear ^1^H–^13^C HSQC (Figure 2D) experiments. According to the literature data of fucoidan from brown algae [32,33], the signals of DAP4 were assigned in Table 3. Most of the protons of the different linkage patterns in the HSQC spectrum were assigned, and because some of the proton signals overlapped, it was difficult to mark them in the spectrum.

The anomeric proton signal of residue A at 5.57 ppm correlated to the anomeric carbon signal at 100.08 ppm, while residue B at 5.50 ppm correlated to the C-1 signal at 95.23 ppm. Thus, the residues A and B were assigned to →4)-α-l-Fuc*p*(2SO_4_)-(1→, →3)-α-l-Fuc*p*(4SO_4_)-(1→ due to the downfield shifts of 82.29, 80.21/82.12, and 75.76 ppm at C-2, C-4, and C-3 ppm, respectively. The H-1 signal at 5.44 ppm of residue C correlated with the C-1 resonance at 97.79 ppm was attributed to disubstituted residue →2)-α-l-Fuc*p*-(1→ for the downfield shift at 82.29 ppm of C-2. The H-1 signal at 5.34 ppm of residue E correlated with the C-1 resonance at 99.20 ppm was attributed to disubstituted residue →3)-α-l-Fuc*p*-(1→ for the downfield shift at 77.94 ppm of C-3. Besides, the H-1 signals at 5.34 ppm of residue F and 5.16 ppm of residue G correlated to C-1 at 97.79 and 99.20 ppm were attributed to disubstituted residues →3,4)-α-l-Fuc*p*-(1→ and →4)-α-l-Fuc*p*-(1→ according to the downfield shifts at 77.94 and 78.32/79.95 ppm at C-3 and C-4 ppm, respectively.

The structural sequences analysis of different linkage patterns was assigned from the ^1^H-^1^H NOESY (Figure 2E) spectra. The H-1 of residue B showed correlation with H-4 of residue A, H-1 of C with H-4 of F, H-1of E with H-3 of E/F, and H-1 of G with H-3 of residues E and F. From these results, it could be deduced that the presence of sequences as follows: →3)-α-l-Fuc*p*(4SO_4_)-(1→4)-α-l-Fuc*p*(2SO_4_)-(1→, →2)-α-l-Fuc*p*-(1→4)-α-l-Fuc*p*-(1→, →3)-α-l-Fuc*p*-(1→3)-α-l-Fuc*p*-(1→, →3)-α-l-Fuc*p*-(1→3,4)-α-l-Fuc*p*-(1→, →4)-α-l-Fuc*p*-(1→3)-α-l-Fuc*p*-(1→ and →4)-α-l-Fuc*p*-(1→3,4)-α-l-Fuc*p*-(1→. Therefore, the results of methylation analysis and NMR showed that the fucoidan DAP4 from *D. antarctica* was not a regular polysaccharide.

According to previous studies, fucoidan derived from brown algae might consist of a similar backbone, which was alternating α-(1–3)- and α-(1–4)-l-fucose residues or repeated α-(1–3)-l-fucose residues. However, there were still many differences between the fucoidan from different materials, such as the sulfate group, side chains, sugar composition, and acetyl groups.

**Figure 3:**
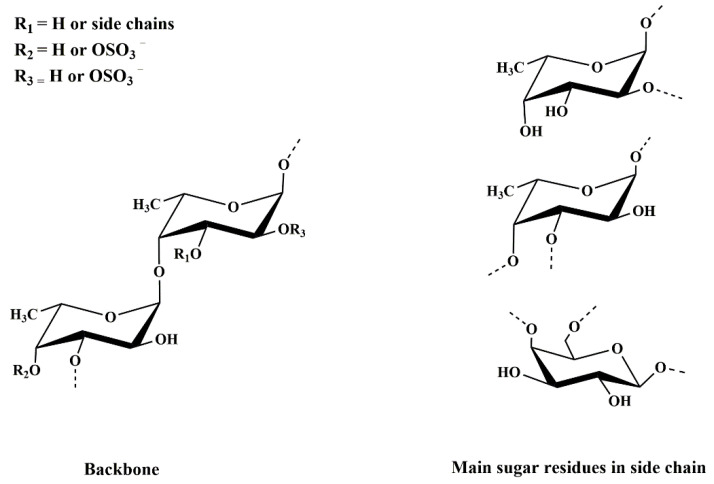
Tentative structure of DAP4 from *Durvillaea antarctica*.

After a comprehensive analysis of spectral information, the primary structure of DAP4 was elucidated. As shown in Figure 3, DAP4 was a branched fucoidan, its backbone mainly consisted of →3)-α-l-Fuc*p*-(1→, →3,4)-α-l-Fuc*p*-(1→ and →4)-α-l-Fuc*p*-(1→ residues with partial sulfation at the C-3 of →4)-α-l-Fuc*p*-(1→, C-2 of →4)-α-l-Fuc*p*-(1→ and C-6 of →4)-β-d-Gal*p*-(1→. Its branches might contain fucose, galactose with or without sulfate ester. Similarly, the fucoidan(F4) from the *D. antarctica* reported by Yang, et al. [22] was mainly composed of fucose, galactose, and glucose in a molar ratio of 26.4:7.1:1.0. The backbone of F4 was composed of α-(1→3) and α-(1→4)-l-fucose residues with sulfate group at C-4 or C-2 positions and branched with α-l-fucose, β-d-galactose, and β-d-glucose residues. Until now, the structure information of fucoidan derived from the genus *Durvillaea* was seldom found. Matsuhiro et al. reported a sulfated fucose-containing polysaccharide containing 55.8% carbohydrate, 23.5% sulfate, and 4.22% uronic acids [34]. In contrast, the fucoidan from *Cladosiphon okamuranus* have a simply linear chain of α-(1→3)-l-fucose residues with sulfate groups at C-4 position [35]. The fucoidan from *Sargassum horneri* has a backbone consisting of alternating α-(1–3)- and α-(1–4)-l-fucose residues, however, the sulfate group mainly at C-3, and less at C-2 position, with side chain at C-4 of α-l-Fuc*p*-(1→2)-α-l-Fuc*p*-(1→residues [29]. The differences in structure, composition, molecular weight and sulfation of such polysaccharides might depend mainly on the following factors: the growth geographical environment, collection season, breeding conditions, and extraction method of the brown seaweed [36,37].

Fucoidan structures from various brown seaweeds have been studied in detail over the past two decades, and in general, have relatively similar and complex structures. They are usually divided into two types according to their backbones: alternating (1–3)- and (1–4)-l-fucose residues, or (1–3)-linked l-fucose residues. For both backbones, they possessed different sulfation patterns with one or two sulfate groups on C-2, C-4, or sometimes C-3 positions [38]. The bioactivity of brown seaweed fucoidan is closely related to their structural characteristics, especially their backbones and sulfation patterns [39]. The fucoidan DAP4 had different structure from other fucoidan from seaweed. The sulfate substitutions were located at the C-4 of→3)-α-l-Fuc*p*-(1→, C-2 of →4)-α-l-Fuc*p*-(1→ and minor C-6 of →4)-β-d-Gal*p*-(1→; besides, the xylose existed only in the form of β-d-Xyl*p*-(1→. Especially, the terminal β-d-xylosyl and →4)-β-d-Gal*p*-(1→ residues were infrequently found in the fucoidan from Durvillaeaceae species [19,22,40].

### 2.3. Immunomodulatory Activity

#### 2.3.1. Effects of DAP4 on RAW264.7 Cell Viability

**Figure 4:**
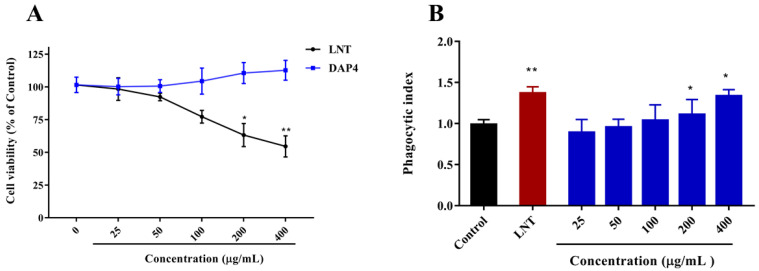
Effects of DAP4 on RAW264.7 cell viability (**A**), and neutral red phagocytic (**B**). * *p* < 0.05, ** *p* < 0.01 versus the control group.

MTT assay was used to investigate the effect of DAP4 on the cell viability of RAW264.7 macrophages. As shown in Figure 4A, DAP4 was not cytotoxic to RAW264.7 cells with a concentration range from 25 to 400 μg/mL. DAP4 could promote cell proliferation at 200 and 400 μg/mL, compared with the control group. In contrast, LNT was detected to show significant cytotoxicity when the concentration was higher than 200 μg/mL (*p* < 0.05). As a result, five distinct dosages of 25, 50, 100, 200, and 400 μg/mL were finally employed to evaluate the immune-enhancing activities of DAP4.

#### 2.3.2. Effect of DAP4 on the Neutral Red Phagocytic Activity

Increased phagocytic activity was one of the most significant features of macrophage activation [41,42]. Accordingly, RAW 264.7 macrophages were pretreated with DAP4 at different concentrations, and their phagocytic activity was determined by the neutral red uptake method. As shown in Figure 4B, DAP4 could significantly enhance the phagocytic activity of RAW 264.7 cells in a dose-dependent manner (*p* < 0.05), as compared to the control group. Notably, the stimulatory effect of DAP4 on the phagocytic activity of RAW 264.7 macrophages was similar to that of LNT with the concentration at 400 μg/mL.

#### 2.3.3. Effect of DAP4 on Lymphocyte Proliferation

**Figure 5:**
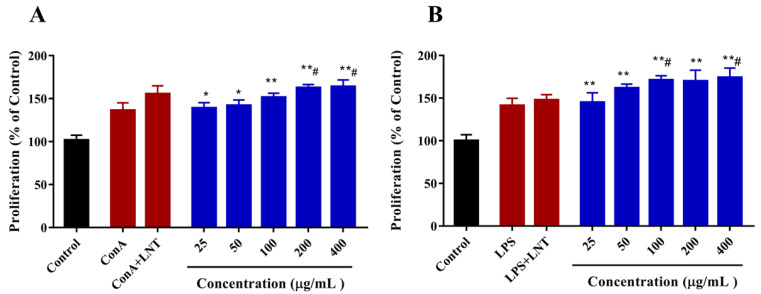
Effects of DAP4 on splenic T lymphocyte proliferation (**A**), splenic B lymphocyte proliferation (**B**). * *p* < 0.05, ** *p* < 0.01, versus with control group; # *p* < 0.05, versus the ConA or LPS groups.

The splenic lymphocytes play important roles in the cellular and humoral immune responses against the antigens in the blood. As lipopolysaccharide (LPS) could trigger the proliferation of B cells, and concanavalin A (ConA) could induce differentiation into T cells [43]. The effect of DAP4 on lymphocyte proliferation was investigated by MTT assay in the present work. As shown in Figure 5A, DAP4 could significantly enhance the proliferation of T lymphocytes stimulated with Con A (*p* < 0.05), the potential effect on T lymphocyte proliferation was comparable to that of LNT. Meanwhile, it was also observed that DAP4 could also improve the proliferation of B lymphocytes stimulated with LPS in a dose-dependent manner (Figure 5B), and the enhancing action of DAP4 was significantly stronger than that of LNT at different concentrations from 50 to 400 μg/mL. Our results indicated that the novel fucoidan DAP4 from *D. antarctica* could promote both T-cell–associated and B-cell-associated lymphocyte proliferation. Additionally, the results further confirmed that DAP4 has an ideal immunostimulatory activity.

#### 2.3.4. Effect of DAP4 on the Production NO

**Figure 6:**
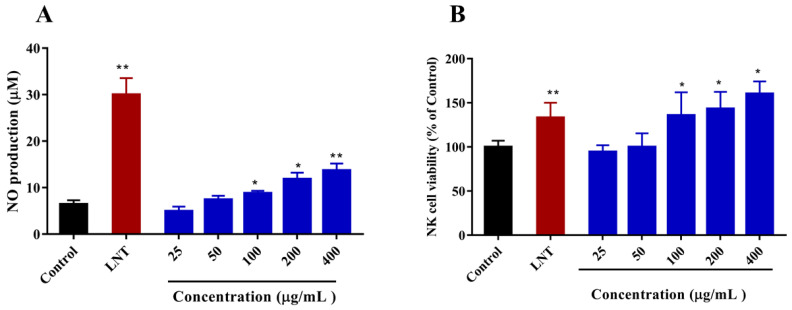
Effects of DAP4 on (**A**) NO production of RAW264.7 macrophages, and (**B**) NK cell viability. All values are expressed as means ± SD of three independent experiments. * *p* < 0.05. ** *p* < 0.01 compared with the control group.

NO is a vital bioactive molecule found throughout the body, its production is a critical component of the mammalian innate immune response [44,45]. To further compare the ability of DAP4 to cause NO production in macrophages, RAW264.7 cells were stimulated with DAP4 (25, 50, 100, 200, and 400 μg/mL), LPS (1 μg/mL), and LNT (100 μg/mL), respectively. As shown in Figure 6A, both polysaccharides increased NO production in RAW 264.7 macrophages dose-dependently (*p <* 0.05). NO productions of cells treated with DAP4 were higher than their production by control cells.

#### 2.3.5. Effect of DAP4 on NK Cells Activity

NK cells are a very important part of the innate immune system, contributing to both anti-viral and anti-tumor immune responses [46]. MTT assay was used to determine the effect of DAP4 on the activity of NK cells. As shown in Figure 6B, DAP4 could significantly enhance NK cell viability at concentrations of 200 and 400 μg/mL compared with the control (*p* < 0.05), suggesting that enhancing NK cells viability is one of the ways DAP4 exerts immunomodulatory activity. Moreover, DAP4 showed stronger enhancing activity of NK cells than that of LNT at concentrations of 200 or 400 μg/mL.

From all the above results, DAP4 exhibited excellent immunomodulatory activity by promoting the proliferation of RAW 264.7 cells, promoting the proliferation of spleen T lymphocytes and B lymphocytes, increasing the production of NO and enhancing the phagocytic ability of macrophages. Besides, DAP4 could also enhance the activity of NK cells. Our findings may provide a basis for using the fucoidan from *D. antarctica* as an immunomodulator for pharmaceutical and food additive applications.

As previous studies reported, the mechanisms of sulfated polysaccharides exerting the immunomodulatory activity are related to the regulation of macrophage function, NK cells, and T/B lymphocytes, in addition to the stimulation of the immune responses of lymphocytes and the activation of the complement system [6]. Han et al. reported a fucoidan from *Fucus vesiculosus* possessing immunomodulatory activity because it could significantly increase the growth of B and T cells in a concentration-dependent manner [47]. Besides, the fucoidan from *Kjellmaniella crassifolia* and *Undaria pinnatifida* were also proved to have excellent immunomodulatory activity by stimulating macrophage cell proliferation and enhancing the secretion of various cytokines, such as granulocyte-macrophage colony-stimulating factor (GM-CSF) and TNF-α [48]. Taken together, fucoidan plays a crucial role in regulating our immune system, the action or mechanism is a complex process that might be regulated by many factors. Thus, the link between the immunomodulatory mechanisms and the structure characteristics of fucoidan requires further exploration.

## 3. Materials and Methods

### 3.1. Materials

Dry brown algae *(Durvillaea antarctica)* was provided by Gather Great Ocean Algae industry group CO., LTD (Qingdao, China). The raw material was milled into a powder and stored in a dry environment at room temperature. Q Sepharose Fast Flow and Sephacryl S-300/HR were purchased from GE Healthcare Life Sciences (Piscataway, NJ, USA). Pullulan standards for HPGPC analysis were purchased from Showa Denko K.K. (Tokyo, Japan). Monosaccharide standards for sugar composition analysis were purchased from Sigma (St. Louis, MO, USA). Pyromellitic acid (PMA), sodium fluoride (NaF), methyl iodide (CH_3_I), NaH, and KBr were also obtained from Sigma. Other materials, such as 732 cation-exchange resin, pyridine, and dimethylsulfoxide (DMSO), were from Signopharm Chemical Reagent CO., LTD (Shanghai, China).

### 3.2. Isolation and Purification of the Sulfated Polysaccharide

The isolation of crude polysaccharide was according to the method of Li, et al. [49]. Briefly, the powder of *D. antarctica* (500 g) was defatted by 95% ethanol with a ratio of 1:5 (*w*/*v*). Then the powder of *D. antarctica* was extracted with distilled water (5 L) at 70 °C for 3 h, the extraction was centrifuged at 5000 rpm for 10 min. This step was repeated three times. The supernatant was merged and concentrated under reduced pressure; next, the supernatant was dialyzed with cellulose membrane (molecular weight cut-off 3.5 kDa) against distilled water at room temperature. The recovered fraction was concentrated and precipitated by 95% ethanol, the final concentration of ethanol was 75%. The precipitate was washed with 100% ethanol and acetone to remove excess moisture, then the crude polysaccharide was dried at 40 °C. Finally, the dry polysaccharide was dissolved in distilled water to an appropriate concentration, mixed with 3 M CaCl_2_, then centrifuged at 5000 rpm for 10 min. The supernatant was lyophilized to obtain a crude fucoidan, designed as DAP.

The crude fucoidan DAP was separated by a Q Sepharose Fast Flow column (30 cm × 3.5 cm) and eluted with a step-wise gradient of NaCl (0, 0.5, 1.0, and 1.5 M). The fraction eluted with 1.5 M NaCl was collected, further purified on a Sephacryl S-300/HR column (100 cm × 2.5 cm), and eluted with 0.2 M NH_4_HCO_3_. The major polysaccharide fractions were pooled, desalted, freeze-dried, and named as DAP4.

### 3.3. Composition Analysis

The phenol-sulfuric acid method was used to determine the total sugar content using fucose as the standard [50]. Protein content was determined by the method of Bradford [51]. Sulfate ester content was measured by the barium rhodizonic acid method [52]. Uronic acid content was estimated by the carbazole-sulfuric acid method using glucuronic acid as the standard [53].

Reversed-phase high performance liquid chromatography (HPLC) after pre-column derivatization was used to analyze the monosaccharide composition [26,54]. Briefly, polysaccharide was hydrolyzed with 2 M trifluoroacetic acid at 105 °C for 6 h, then the dry hydrolysate was dissolved in 100 μL of distilled water and derivatized with 120 μL of 1- phenyl-3-methyl-5-pyrazolone (PMP) in methanol. The mixture was added to 100 μL of 0.3 M HCl solution and vigorously shaken and centrifuged. The supernatant was filtered through 0.22 μm nylon membranes, and the resulting solution was injected into the XDB-C18 column (4.6 mm × 250 mm). The chromatogram was performed on an Agilent 1260 Infinity HPLC instrument fitted with an Agilent XDB-UV detector (254 nm, Palo Alto, USA). The mobile phase was a mixture of 0.1 M KH_2_PO_4_ (pH 6.7)-acetonitrile (83:17). The flow rate was 1.0 mL/min and column temperature was 30 °C. Identification of sugar was done by comparison with retention time of reference sugars (l-rhamnose, l-arabinose, d-xylose, l-fucose, d-mannose, d-galactose, d-glucose, d-glucuronic acid, d-galacturonic acid, d-glucosamine). The sugar content was calculated by the ratio of the peak areas of sugar and correspondent reference sugar [54].

### 3.4. Purity and Molecular Weight

Purity and molecular weight of polysaccharide were assessed by high performance gel permeation chromatography (HPGPC) [54]. The assay was performed on an Agilent 1260 Infinity HPLC instrument fitted with a Shodex OHpak SB-804 HQ column (8.0 mm × 300 mm, Tokyo, Japan) and elution with 0.2 mol/L Na_2_SO_4_ at a flow rate of 0.5 mL/min. The signals were measured using a refractive index detector (Agilent RID-10A Series). The molecular weight was estimated by reference to a calibration curve made by pullulan standards; molecular weights of pullulan standards were 5.9, 9.6, 21.1, 47.1, 107, 200, 344, and 708 kDa, respectively.

### 3.5. Desulfation and Methylation Analysis

To compare the methylation-analysis data of sulfated polysaccharide with its desulfated form could provide clearer information about the sulfate group. Desulfation was performed as previously described [54]. Briefly, DAP4 (200 mg) was dissolved in water and passed through an ion-exchange column (732 resin, H^+^ form), which was eluted with distilled water. The combined effluent was neutralized with pyridine to pH 9.0, and then lyophilized to give a white powdered pyridinium salt. The product was dissolved in dimethyl sulfoxide (12 mL) containing pyromellitic acid (124 mg) and NaF (120 mg), pyridine (4 mL) was added, and then the solution was shaken at 100 °C for 4 h. After the reaction was completed, the product was dialyzed against distilled water, freeze-dried, and designated as DAP4-Ds. The completeness of desulfation was assayed by sulfate ester content of the polysaccharide.

Methylation analysis was performed according to the method of Hakomori with some modifications [55]. Polysaccharide (2 mg) was dissolved in DMSO (0.6 mL) and anhydrous NaH (100–200 mg) was then added and the mixture was stirred under N_2_ at 60 °C for 1.5 h. Methyl iodide was then added to the mixture and stirred for a further 1.5 h. The reaction was terminated by adding 1 mL distilled water, and the residue was extracted with CHCl_3_. The extract was washed with distilled water and evaporated to dryness. The completion of methylation was confirmed by IR spectroscopy as the disappearance of −OH bands. Methylated samples were hydrolyzed with 2 M trifluoroacetic acid at 105 °C for 6 h. The methylated products were converted into their corresponding alditols by reduction with NaBD_4_ and acetylated. Thereafter, partial methylated alditol acetates were analyzed by gas chromatography–mass spectrometry (GC–MS) on a TRACE 1300-ISQ instrument (Thermo Scientific, Waltham, MA, USA) fitted with a DB 225 fused silica capillary column (0.25 mm × 30 mm, Agilent Technologies, Santa Clara, CA, USA). The partially methylated alditol acetates were identified according to the Complex Carbohydrate Structural Database of the Complex Carbohydrate Research Centre (https://glygen.ccrc.uga.edu/, accessed on 21 September 2021).

### 3.6. Spectroscopy Analysis

FTIR spectra of DAP4 were measured on a Nicolet Nexus 470 spectrometer (Thermo Fisher Scientific, Waltham, MA, USA). DAP4 and DAP4-Ds (2.0 mg) were mixed with KBr powder, ground, and pressed into a 1 mm pellet for FTIR measurements, KBr blank film was used as a control group. The frequency range was 4000–400 cm^−1^ and scanning number was 32 times.

NMR analyses of DAP4 was performed on an Agilent DD2 500 MHz NMR spectrometer (Agilent Technologies, Santa Clara, CA, USA). The temperature was 25 °C. Briefly, DAP4 (50 mg) was dissolved in 500 μL of D_2_O and freeze-dried. The operation was repeated three times. Finally, DAP4 was redissolved in 500 μL D_2_O and transferred into NMR tube. The internal standard was acetone (^1^H 2.225 ppm, ^13^C 31.07 ppm). Spectra were processed and analyzed by MestReNova (V12.0.3, Mestrelab Research, Spain).

### 3.7. Immunomodulatory Activity

#### 3.7.1. Cell Cultures

RAW264.7 cells, a line from murine macrophages were from ATCC (Manassas, VA, USA, CVCL_0493). RAW264.7 cells were cultured in RPMI-1640 medium supplemented with 10% (*v*/*v*) FBS. Besides, penicillin (100 U/mL) and streptomycin sulfate (100 μg/mL) were added to the medium. Cells were incubated at 37 °C in a humidified incubator with 5% CO_2_. Before plating, cells were digested by 0.25% pancreatin and washed with PBS, cells were plated on 6- or 96-well plates for the following experiments.

#### 3.7.2. Cytotoxicity Assay

Cytotoxicity of DAP4 was measured by MTT assay. Briefly, RAW264.7 cells were seeded on 96-well plates. After incubating at 37 °C for 24 h, cells were exposed to different concentrations of DAP4 (25, 50, 100, 200, and 400 μg/mL) and lentinan (LNT) for 24 h at 37 °C. After that time, 20 μL of MTT solution (5 mg/mL in PBS) was added to each well, and incubated for 4h. Finally, the medium was removed and 150 μL DMSO was added to dissolve the formazan salt. The absorbance was tested at 570 nm on a Bio-Rad model 680 Microplate Reader (Hercules, CA, USA).

#### 3.7.3. Neutral Red Phagocytosis Assay

The pinocytosis activity of RAW264.7 cells was measured by neutral red uptake assay according to the method of Yang et al. [21]. Briefly, RAW264.7 cells were seeded on 96-well plates, after incubating at 37 °C for 24 h, cells were exposed to different concentrations of DAP4 (25, 50, 100, 200, and 400 μg/mL), and LNT (1–100 μg/mL) for 24 h. After that time, the cell culture medium was removed and 100 μL of 0.075% neutral red was added into each well. After being incubated at 37 °C for 0.5 h, cells were washed with PBS three times. Furthermore, 150 μL of cell lysate containing of acetic acid and ethanol (*v*/*v*, 1:1) were added to each well and incubated at 4 °C for another 0.5 h. Finally, the absorbance was tested on the Bio-Rad model 680 Microplate Reader at 570 nm.

#### 3.7.4. NO Production Assay

NO production was measured by Griess assay according to the method of Zhao et al. [56]. Briefly, RAW264.7 cells were seeded on 96-well plates and incubated at 37 °C for 12 h, cells were treated with LPS (1 μg/mL) or DAP4 (25, 50, 100, 200, and 400 μg/mL) at 37 ℃ for 24 h. After that, the supernatant was collected and used to measure the content of NO in the supernatant. The supernatant (50 μL) was added to the mixture of 50 μL Griess reagent I (1% sulfanilamide dihydrochloride) and Griess reagent II (0.1% naphthyl ethylenediamine dihydrochloride) at the ratio of 1:1. After being incubated at room temperature for 0.5 h, the absorbance at 540 nm was measured on the Bio-Rad model 680 Microplate Reader. NaNO_2_ was used as a standard, cells without any treatment were used as the negative control while LPS-treated cells were a positive control.

#### 3.7.5. Lymphocyte Proliferation Assay

The splenocyte proliferation was evaluated by MTT-based colorimetric assay as previously described [57]. Briefly, the spleens of BALB/c mice (male, 8–10 weeks old) were removed aseptically. Spleen cells of mice were obtained by gently teasing the organ in RPMI-1640 medium under aseptic condition and centrifuged at 1000 rpm for 10 min. Splenic T- and B-lymphocyte proliferation was induced with the mitogens ConA and LPS, respectively [58]. Hemolytic Gey’s solution was used to remove the red blood cells. Then spleen cells were resuspended in complete RPMI 1640 medium and were plated on 96- well culture platers with ConA (2.5 μg/mL) or LPS (10 μg/mL). After the cells were cultured with or without DAP4 (25, 50, 100, 200, and 400 μg/mL) at 37 °C in a humidified atmosphere containing 5% CO_2_ for 48 h, 20 μL MTT (5 mg/mL) were added to each well and incubated for another 4 h. Finally, the medium was removed, 150 μL DMSO was added to dissolve the formazan salt. The absorbance was tested at 570 nm on a Bio-Rad model 680 Microplate Reader.

#### 3.7.6. NK Cells Activity Assay

The cytolytic activity of NK cells was detected by a standard ^51^Cr release assay according to the method of Zhao et al. [56]. Briefly, 100 μL of splenocytes were seeded into 96-well plate and used as effector cells, then 100 μL of LNT was added to a final concentration of 50 μg/mL. Furthermore, 100 μL of RPMI-1640 medium was added and used as control group, or 100 μL of DAP were added. Then the cells were incubated at 37 °C in a 5% CO_2_ incubator. The target cells, YAC-1 from ATCC (Manassas, VA, USA, CVCL_2244), were added at an effector-to-target at a ratio of 10:1. After incubation for another 4 h, 20 µL of MTT (5 mg/mL) was added, and the cells were also incubated for 4 h. The medium was removed and 150 μL DMSO was added to dissolve the formazan salt. The absorbance was tested at 570 nm on a Bio-Rad model 680 Microplate Reader. The cytotoxicity of NK cells was calculated using the following formula: NK cell cytotoxicity (%) = 1 − (OD_2_ − OD_3_) / OD_1_ × 100% (OD_1_: OD value of target cells control; OD_2_: OD value of test group; OD_3_: OD value of the effector cells group).

### 3.8. Statistical Analysis

All bioassay values were expressed as means ± standard deviation (SD). At least three samples were prepared for assays of every attribute. The experimental data were subjected to one-way ANOVA test (GraphPad Prism 7.00, La Jolla, CA, USA). Statistical significance was denoted by asterisks and hashes. * and # represented *p* < 0.05, while ** and ## were *p* < 0.01.

## 4. Conclusions

In conclusion, a fucoidan DAP4 was extracted, purified, and characterized from the brown seaweed *D. antarctica*. The backbone of DAP4 consists of (1→3)-α-l-Fuc*p* and (1→4)-α-l-Fuc*p* residues with a small degree of branching at C-3 of (1→4)-α-l-Fuc*p* residues, which contained fucose, galactose with or without sulfate ester. About 22.30% of the total number of sugar residues in DAP4 was substituted by sulfate groups, and sulfate groups were at C-3 of (1→4)-α-l-Fuc*p*, C-2 of (1→4)-α-l-Fuc*p* and minor C-6 of (1→4)-β-d-Gal*p*. Immunomodulatory activities in vitro demonstrated that DAP4 exhibited excellent immunomodulatory activity by promoting the proliferation of RAW264.7 cells, promoting the proliferation of spleen lymphocytes, exceeding NO production, and enhancing phagocytic of macrophages. Besides, DAP4 could also produce better enhancement on the vitality of NK cells. DAP4 had high immune-enhancing activity and might be a potential source of immunomodulatory fucoidan with a novel structure. Further analysis on the structure–activity relationship of the marine algal fucoidan would play an important role in the understanding of immunomodulatory properties. The detailed mechanism of immunomodulatory action of DAP4 is currently still being investigated.

## Figures and Tables

**Table 1:** Composition analysis of polysaccharide isolated from *D. antarctica*.

Sample	Total Sugar (%)	Protein (%)	Sulfate (%)	Mw (kDa)	Monosaccharide Composition (Molar Ratio %)					
					Man	GlcA	Glc	Gal	Xyl	Fuc
DAP	69.8	3.4	20.7	nd	9.7	11.8	19.7	18.1	5.9	34.8
DAP1	78.2	2.1	4.5	754	9.2	nd ^a^	82.1	3.4	nd ^a^	5.3
DAP2	76.7	1.0	15.6	532	23.5	0.9	23.7	9.5	6.8	35.6
DAP3	82.3	nd	17.7	368	17.8	2.3	4.3	32.1	4.2	39.3
DAP4	75.9	0.2	22.3	92	2.9	1.2	nd ^a^	3.5	4.5	87.9

^a^ not detected.

**Table 2:** Methylation analyses of DAP4 and DAP4-Ds.

Methylate Alditol Acetate	Molar Percent Ratio		Linkage Pattern
	DAP4	DAP4-Ds	
1,5-Di-O-acrtyl-2,3,4-tri-O-methyl-d-xylitol	3.22	3.41	Xyl*p*-(1→
1,5-Di-O-acetyl-2,3,4-tri-O-methyl-l-fucitol	7.68	6.42	Fuc*p*-(1→
1,5-Di-O-acetyl-2,3,4,6-tetra-O-methyl-d-galacitol	1.47	1.13	Gal*p*-(1→
1,3,5-Tri-O-acetyl-2,4-di-O-methyl-l-fucitol	24.73	37.92	→3)-Fuc*p*-(1→
1,4,5-Tri-O-acetyl-2,3-di-O-methyl-l-fucitol	21.42	28.23	→4)-Fuc*p*-(1→
1,2,5-Tetra-O-acetyl-3,4-di-O-methyl-fucitol	5.24	7.54	→2)-Fuc*p*-(1→
1,3,4,5-Tetra-O-acetyl-2-O-methyl-fucitol	24.43	12.01	→3,4)-Fuc*p*-(1→
1,2,4,5-Tri-O-acetyl-3-O-methyl-l-fucitol	8.36	nd ^a^	→2,4)-Fuc*p*-(1→
1,4,5-Tri-O-acetyl-2,3,6-tri-O-methyl-d-galactitol	2.21	3.34	→4)-Gal*p*-(1→
1,4,5,6-Tetra-O-acetyl-2,3-di-O-methyl-d-galactitol	1.24	nd ^a^	→4,6)-Gal*p*-(1→

^a^ not detected.

**Table 3:** ^1^H and ^13^C NMR spectral data of DAP4.

Residue ^b^	Chemical Shifts (ppm) ^a^					
	H1/C1	H2/C2	H3/C3	H4/C4	H5/C5	H6/C6
**A**	5.57/100.08	4.64/82.29	3.96/73.59	4.29/80.21	3.90/68.31	1.43/17.22
**B**	5.50/95.23	4.21/76.15	4.60/75.76	4.71/82.12	3.90/68.31	1.43/17.22
**C**	5.44/97.79	4.29/82.29	4.11/73.69	4.04/70.32	3.68/68.31	1.43/17.22
**D**	5.41/110.16	3.85/73.42	--/--	--/--	--/--	--/--
**E**	5.34/99.20	4.29/73.59	4.11/77.94	3.96/74.71	3.90/68.31	1.30/17.22
**F**	5.34/97.79	4.29/73.59	4.11/77.94	4.23/78.32	4.01/69.7	1.30/17.22
**G**	5.16/99.20	4.29/73.40	4.03/73.59	4.34/79.95	4.01/69.7	1.30/17.22
**H**	4.52/104.70	--/--	--/--	--/--	--/--	3.80/62.33

^a^ Chemical shifts are referenced to internal acetone. ^b^ **A**–**H** correspond to →4)-α-l-Fuc*p*(2SO_4_)-(1→, →3)-α-l-Fuc*p*(4SO_4_)-(1→, →2)-α-l-Fuc*p*-(1→, β-d-Xyl*p*-(1→, →3)-α-l-Fuc*p*-(1→, →3,4)-α-l-Fuc*p*-(1→, →4)-α-l-Fuc*p*-(1→ and β-d-Gal*p*-(1→.

## Data Availability

The data presented in this study are available on request from the corresponding authors.

## Supplementary Materials

The following supporting information can be downloaded at: https://www.mdpi.com/article/10.3390/md20040223/s1, Figure S1: Total ion current (TIC) chromatograms of DAP4 and DAP4-Ds on GC–MS and mass spectra; Figure S2: MS of partial O-methylated alditol acetates from DAP4 and its desulfation form, DAP4-Ds.
