# Supplementary material for: Purification, Chemical Characterization and Immunomodulatory Activity of a Sulfated Polysaccharide from Marine Brown Algae Durvillaea antarctica
Source: Mar Drugs. 2022 Mar 24;20(4):223. doi: 10.3390/md20040223 (PMC9026115; doi:10.3390/md20040223)
Supplement: Supplementary file 1 [file marinedrugs-20-00223-s001.zip › marinedrugs-1626769-supplementary.pdf]

Supplementary material for:

Purification, chemical characterization and immunomodulatory activity of a sulfated polysaccharide from marine brown algae *Durvillaea antarctica*

Ling Qin [1], Hui Xu[1], Yingying He[1,3], Chen Liang[1], Kai Wang [1], Junhan Cao[1], Changfeng Qu[1, 2, 3*], Jinlai Miao [1, 2,3 *]

[1] Key Laboratory of Marine Eco-Environmental Science and Technology, First Institute of Oceanography, Ministry of Natural Resource, Qingdao, 266061, China

[2] Laboratory for Marine Drugs and Bioproducts of Qingdao National Laboratory for Marine Science and Technology, Qingdao, 266237, China

[3] Qingdao Key Laboratory of Marine Natural Products, College of Chemical Engineering, Qingdao University of Science and Technology, Qingdao, 266061, China

*Correspondence: Changfeng Qu; Jinlai Miao; Tel: +86-532-8896-7430 (J.M.)

Email: cfqu@fio.org.cn; miaojinlai@fio.org.cn (J.M.)

SUPPLEMENTARY FIGURES

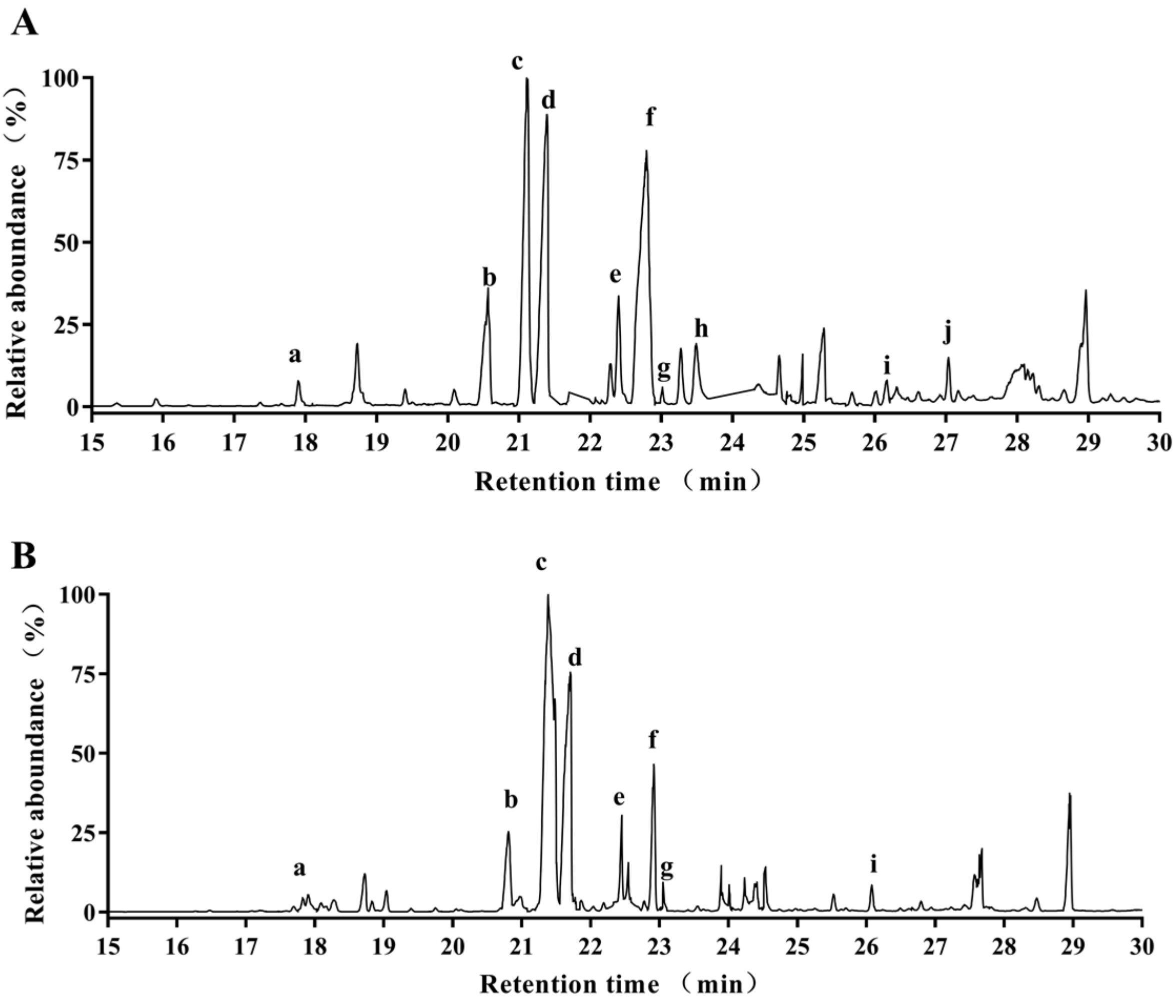

Figure S1. Total ion current (TIC) chromatograms of DAP4 and DAP4-Ds on GC–MS and mass spectra. **(A)** TIC chromatogram on GC–MS of DAP4; **(B)** TIC chromatogram on GC–MS of DAP4-Ds.

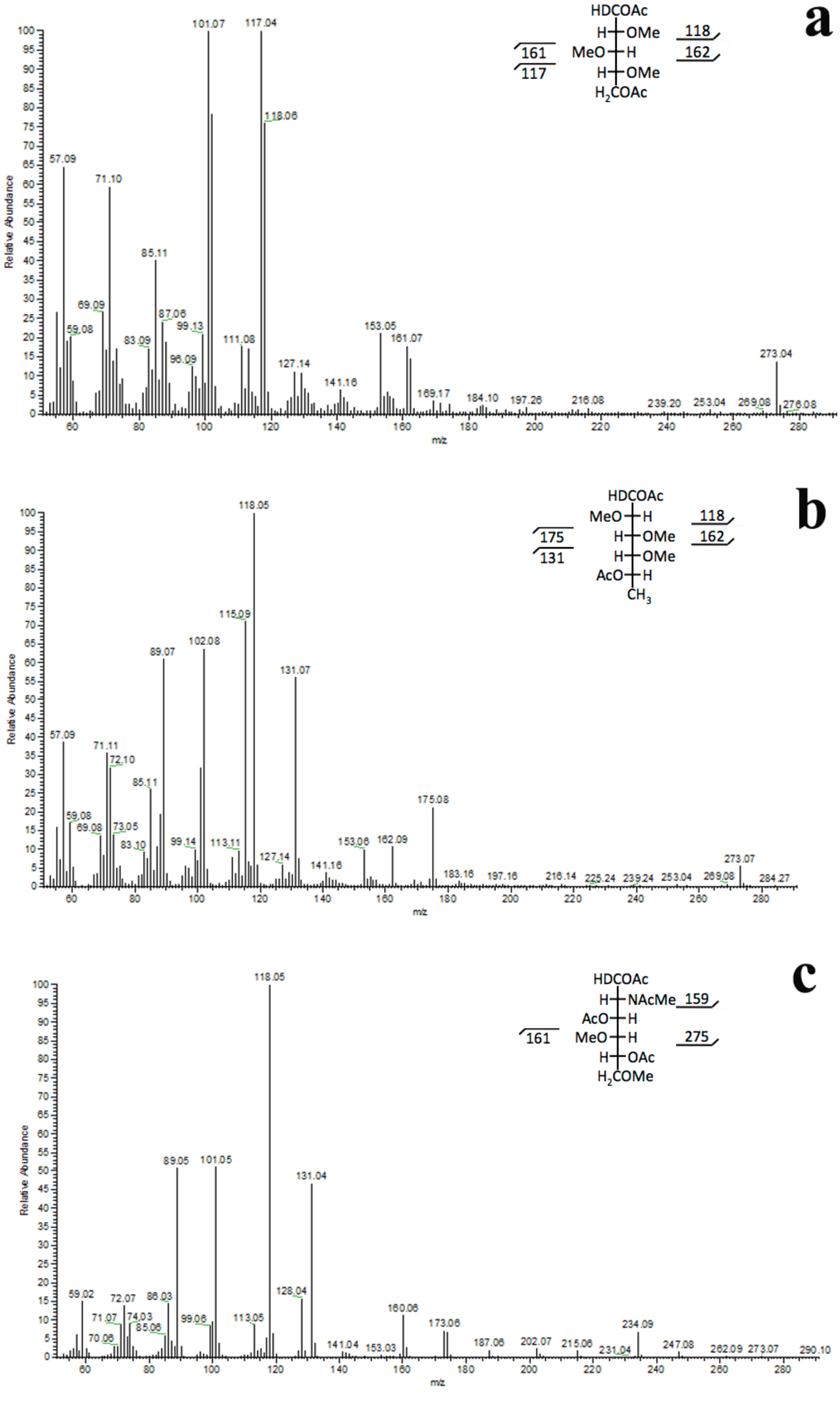
a
HDCOAc
H—OMe
MeO—H
H—OMe
H2COAc
118
162
161
117
Relative Abundance
m/z
57.09
71.10
85.11
69.09
59.08
83.09
87.06
99.13
96.09
101.07
117.04
118.06
111.08
127.14
141.16
153.05
161.07
169.17
184.10
197.26
216.08
239.20
253.04
269.08
273.04
276.08
b
HDCOAc
MeO—H
H—OMe
H—OMe
AcO—H
CH3
118
162
175
131
Relative Abundance
m/z
57.09
59.08
69.08
71.11
72.10
73.05
83.10
85.11
89.07
99.14
102.08
113.11
115.09
118.05
127.14
131.07
141.16
153.06
162.09
175.08
183.16
197.16
216.14
225.24
239.24
253.04
269.08
273.07
284.27
c
HDCOAc
H—NAcMe
AcO—H
MeO—H
H—OAc
H2COMe
159
275
161
Relative Abundance
m/z
59.02
70.06
71.07
72.07
74.03
85.06
86.03
89.05
99.06
101.05
113.05
118.05
128.04
131.04
141.04
153.03
160.06
173.06
187.06
202.07
215.06
231.04
234.09
247.08
262.09
273.07
290.10

d

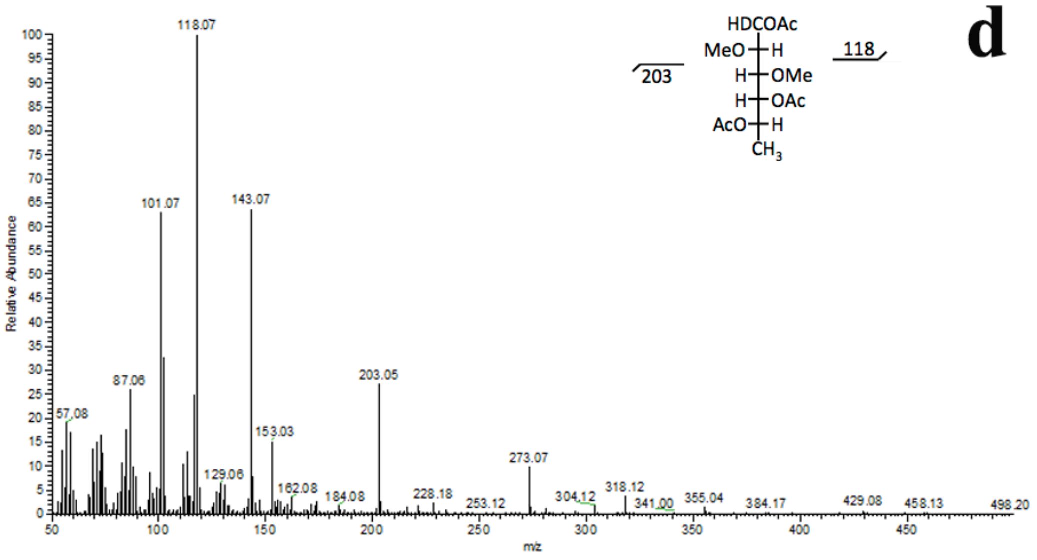
HDCOAc
MeO—H
H—OMe
H—OAc
AcO—H
CH_3
203
118
118.07
101.07
143.07
87.06
57.08
203.05
153.03
273.07
Relative Abundance
m/z

e

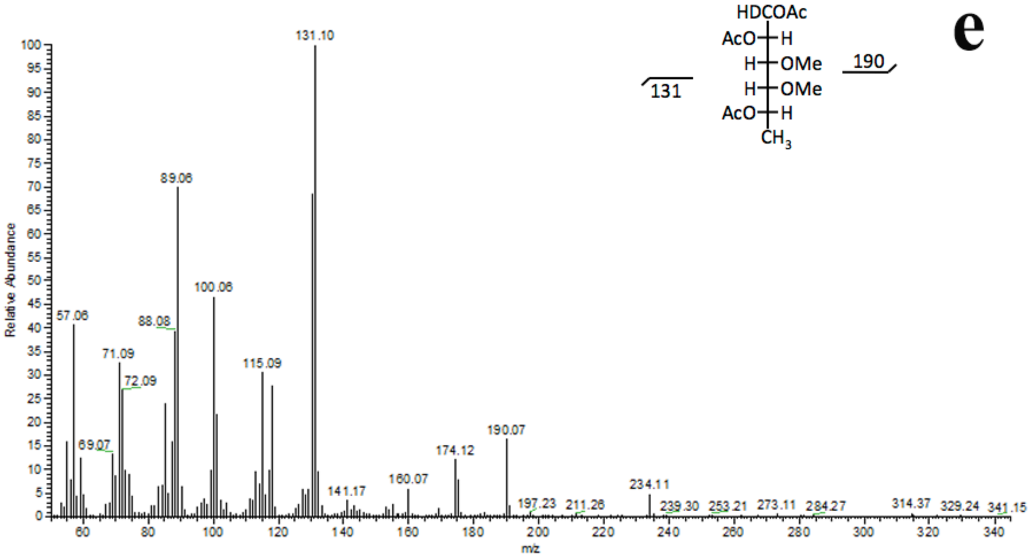
HDCOAc
AcO—H
H—OMe
H—OMe
AcO—H
CH_3
131
190
131.10
89.06
100.06
57.06
88.08
71.09
115.09
190.07
174.12
234.11
Relative Abundance
m/z

f

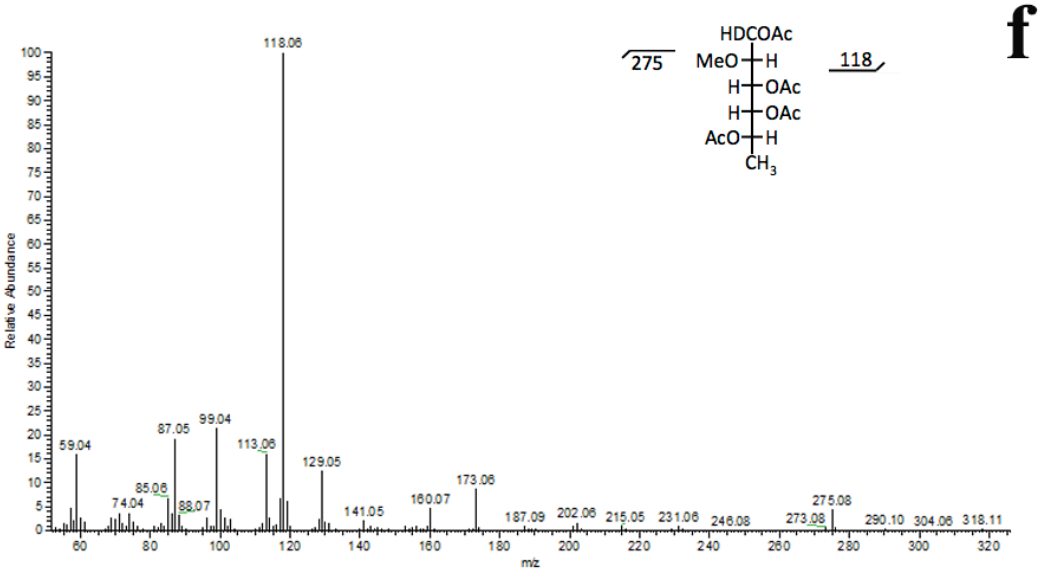
HDCOAc
MeO—H
H—OAc
H—OAc
AcO—H
CH_3
275
118
118.06
99.04
87.05
59.04
113.06
129.05
173.06
275.08
Relative Abundance
m/z

g

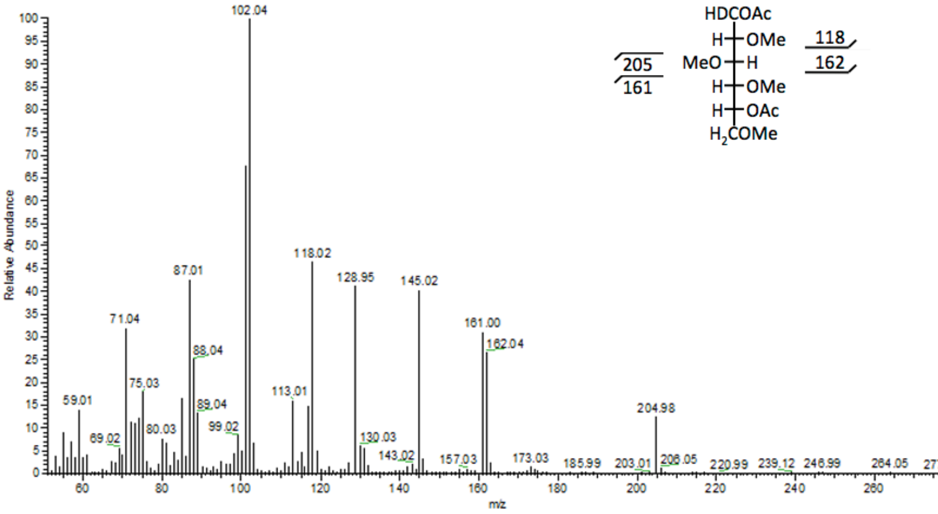
HDCOAc
H–OMe
MeO–H
H–OMe
H–OAc
H_2COMe
118
162
205
161
Relative Abundance
m/z
102.04
118.02
87.01
128.95
145.02
71.04
161.00
162.04
88.04
75.03
113.01
59.01
89.04
204.98
80.03
69.02
99.02
130.03
143.02
157.03
173.03
185.99
203.01
206.05
220.99
239.12
246.99
264.05
277.03

h

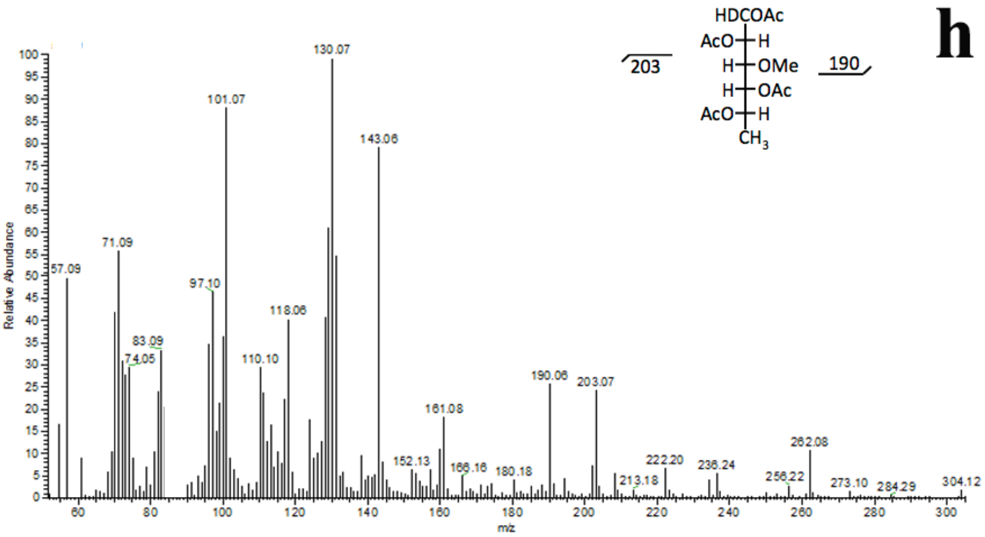
HDCOAc
AcO–H
H–OMe
H–OAc
AcO–H
CH_3
203
190
Relative Abundance
m/z
130.07
101.07
143.06
71.09
57.09
97.10
118.06
83.09
74.05
110.10
190.06
203.07
161.08
262.08
152.13
166.16
180.18
222.20
213.18
236.24
256.22
273.10
284.29
304.12

i

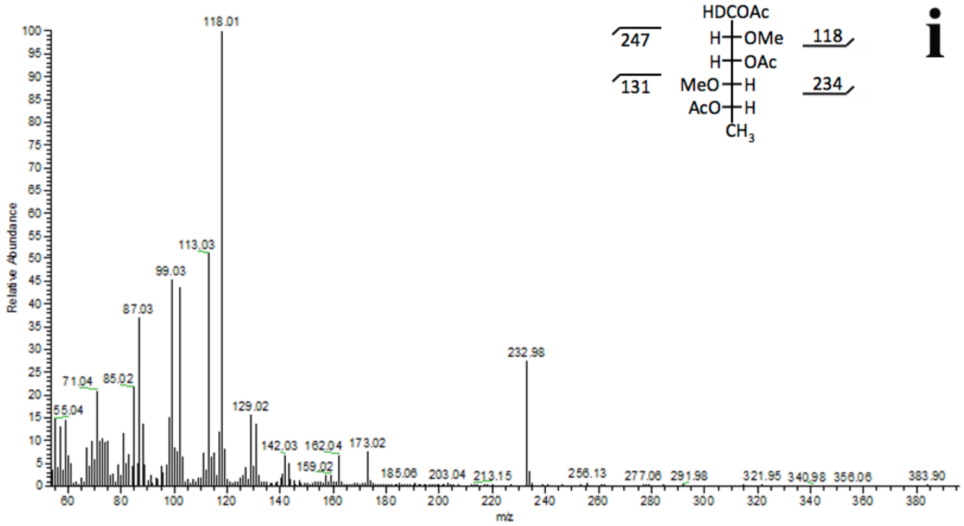
HDCOAc
H–OMe
H–OAc
MeO–H
AcO–H
CH_3
247
131
118
234
Relative Abundance
m/z
118.01
113.03
99.03
87.03
232.98
71.04
85.02
55.04
129.02
142.03
162.04
173.02
159.02
185.06
203.04
213.15
256.13
277.06
291.98
321.95
340.98
356.06
383.90

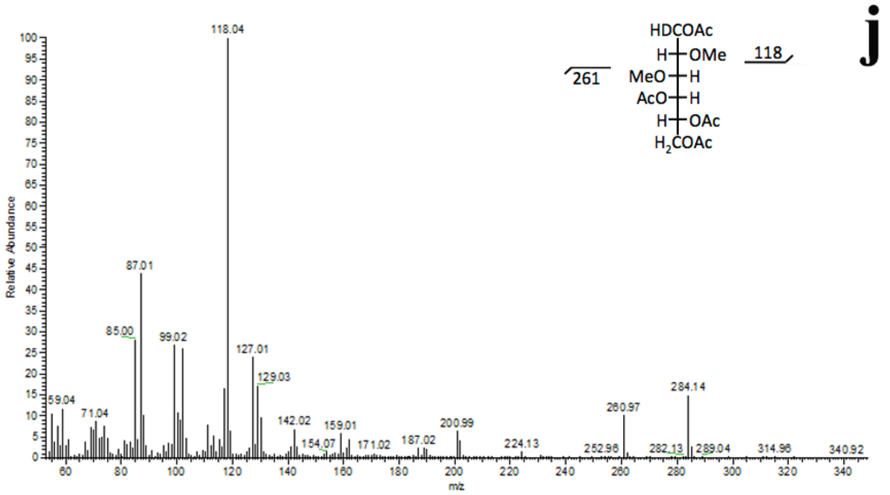

Figure S2. MS of partial O-methylated alditol acetates from DAP4 and its desulfation form, DAP4-Ds.(**a** was MS of 1,5-Di-O-acrtyl-2,3,4-tri-O-methyl-D-xylitol; **b** was MS of 1,5-Di-O-acetyl-2,3,4-tri-O-methyl-L-fucitol; **c** was MS of 1,3,5-Tri-O-acetyl-2,4-di-O-methyl-L-fucitol; **d** was MS of 1,4,5-Tri-O-acetyl-2,3-di-O-methyl-L-fucitol; **e** was MS of 1,2,5-Tri-O-acetyl-3,4-di-O-methyl-L-fucitol; **f** was MS of 1,3,4,5-Tetra-O-acetyl-2-O-methy-L-fucitol; **g** was MS of 1,5-Di-O-acetyl-2,3,4,6-tetra-O-methyl-D- galactitol; **h** was MS of 1,2,4,5-Tetra-O-acetyl-3-O-methy-L-fucitol; **i** was MS of 1,4,5-Tri-O-acetrl-2,3,6-tri-O-methyl-D-galactitol; **j** was MS of 1,4,5,6-Tetra-O-acetyl-2,3-di-O-methyl-D- galactitol)
